# In Situ Regeneration of Si-based ARROW-B Surface Plasmon Resonance Biosensors

**DOI:** 10.1007/s40846-015-0049-0

**Published:** 2015-06-20

**Authors:** Hsin-Feng Hsu, Yen-Ting Lin, Yang-Tung Huang, Ming-Feng Lu, Chyong-Hua Chen

**Affiliations:** Department of Electronic Engineering and Institute of Electronics, National Chiao Tung University, Hsinchu, Taiwan; Department of Biological Science and Technology, National Chiao Tung University, Hsinchu, Taiwan; Department of Electronics Engineering, Minghsin University of Science and Technology, Hsinchu, Taiwan; Department of Photonics, National Chiao Tung University, Hsinchu, Taiwan

**Keywords:** Surface plasmon resonance (SPR), Antiresonant reflecting optical waveguide type B (ARROW-B), Biosensor, Sensor regeneration, Single-step lithography

## Abstract

Si-based antiresonant reflecting optical waveguide type B (ARROW-B) surface plasmon resonance (SPR) biosensors allow label-free high-sensitivity detection of biomolecular interactions in real time. The ARROW-B waveguide, which has a thick guiding layer, provides efficient coupling with a single-mode fiber. The Si-based ARROW-B SPR biosensors were fabricated by using the standard semiconductor fabrication processes with a single-step lithography. A fluid flow system was designed to transport samples or analytes. The waveguide consists of propagation and SPR sensing regions. The propagation regions in the front and rear of the SPR sensing region have a symmetric cladding structure to isolate them from environmental changes. A high-index O-ring is used to seal the liquid flow channel. The intensity interrogation method was used to characterize the sensors. The sensitivity of the biosensors was 3.0 × 10^3^ µW/RIU (refractive index unit) with a resolution of 6.2 × 10^−5^ RIU. An in situ regeneration process was designed to make the sensors reusable and eliminate re-alignment of the optical measurement system. The regeneration was realized using ammonia-hydrogen peroxide mixture solutions to remove molecules bound on the sensor surface, such as self-assembled 11-mercapto-1undecanoic acid and bovine serum albumin. SPR was used to monitor the regeneration processes. The experimental results show that the sensing response did not significantly change after the sensor was reused more than 10 times. In situ regenerations of the sensors were achieved.

## Introduction

Surface plasmon resonance (SPR) biosensors have been extensively investigated due to their advantages, such as high sensitivity to refractive index change on metal surfaces, label-free biomoleculur detection, and real-time detection [1]. The first application of SPR sensors was gas sensing [2]. Since then, SPR biosensors have been widely used in health-related applications, such as medical diagnostics, environmental monitoring, and food safety [3–5].

SPR is a charge-density oscillation that exists at the interface of two media with dielectric constants of opposite signs, such as a metal and a dielectric. The oscillation can be excited by the transverse magnetic (TM)-polarized waves only and creates photon-plasmon surface electromagnetic waves, called surface plasmon waves (SPWs), at the metal/dielectric interface.

The propagation constant *k*_SPW_ of SPWs at the interface is [1]:

1 $$ k_{SPW} = k_{0} \sqrt {\frac{{\varepsilon_{m} \times \varepsilon_{d} }}{{\varepsilon_{m} + \varepsilon_{d} }}} $$

where *k*_0_ is the free-space wave number of the optical wave and *ε*_m_ (=*ε*_mr_ + *iε*_mi_) and *ε*_d_ are the dielectric constants of the metal and dielectric layers, respectively. When the propagation constant β of the input optical wave along the propagation direction at the interface is equal to *k*_SPW_, the resonance condition is satisfied, which is called SPR.

In most conditions, *ε*_mr_ < −*ε*_d_, i.e., the momentum of SPWs is higher than that of the optical wave, SPWs cannot be excited directly. Using attenuated total reflection in prism coupler structures is one method for enhancing momentum [2]. In this method, the optical wave is totally reflected at the interface between a prism and a metal layer, which evanescently penetrates through the metal layer to satisfy the SPR condition and excite an SPW at the outer boundary of the metal layer. The excitation of an SPW results in a drop in the intensity of the reflected light and can be observed as a dip in the angular or wavelength spectrum of the reflected light [5]. Since the electromagnetic field is mostly distributed in the dielectric layer, the SPR condition highly depends on the property of the dielectric layer. Therefore, SPR is sensitive to the refractive index variation of the dielectric layer.

A waveguide-based structure is another method for enhancing the momentum of the optical wave to excite SPWs [1]. The propagation constant of the guided optical wave (β_WG_) can be designed to match that (*k*_SPW_) of the SPW. SPR can be monitored by measuring the intensity of the optical wave near the resonance.

For integrated optics, waveguides are usually coupled with fiber at the input/output interface. It is important to choose a waveguide material with a low propagation loss and good compatibility with fibers. Because the evanescent wave profile varies with guided modes and the excited mode number strongly depends on the alignment of a multimode waveguide and a fiber, a single-mode waveguide is preferred for biochemical sensors. However, the core sizes of conventional waveguides are much smaller than those of single-mode fibers. In addition, very thick cladding layers are necessary to achieve low propagation loss. It is thus difficult to realize a waveguide-based SPR sensor using conventional waveguides. A novel integrated optical waveguide structure, called an antiresonant reflecting optical waveguide (ARROW), has been proposed [6]. Compared with a conventional waveguide, ARROW has the following advantages: single-mode transmission, relatively large core size, which is suitable for efficient connection to a single-mode fiber, flexible design rules for choosing optical materials and thickness, and compatibility with standard semiconductor fabrication process. However, ARROW supports transverse electric (TE) waves only, which is inadequate for our SPR sensor. On the other hand, a type-B antiresonant reflecting optical waveguide (ARROW-B) is a modified ARROW structure that can guide both TE and TM waves [7]. Here, the ARROW-B is utilized to support the low-loss TM-polarized transmission required to excite SPWs at the interface between the metal and dielectric layers.

Molecular recognition plays an important role in biosensing, so specific bindings such as antigen–antibody binding and receptor-ligand binding are required. Both physical and chemical adsorption, via Langmuir–Blodgett (LB) films and self-assembled monolayers (SAMs), respectively, can immobilize biomolecules onto a metal surface (e.g., Au, Ag, and Cu) [8]. Because SAMs contain a thiol group to bond with atoms of the metal through covalent bonding, they are more uniform and stable than LB films. Moreover, SAMs provide a convenient and flexible way to generate thin and well-ordered biological molecular monolayers. Most importantly, SAMs provide a variety of functional groups at the terminal site, leading to different interfacial properties for linking with various biomolecules [9]. The most commonly used metal for exciting SPWs is gold owing to its inertness, stability in aqueous environments, and good biocompatibility.

In many biochemical sensing applications, the transducer functionalized with SAMs is normally discarded after use because of the difficulty in removing the thiols from the gold surface. It is thus desirable to develop approaches to remove these biomolecules from a gold surface for sensor reuse. The reuse of sensors reduces cost and allows better experimental controls since the characteristics of the biochip would remain the same. Many methods have been proposed for removing thiols from a gold surface, including electrochemical cleaning [10], ultraviolet/ozone exposure [11], and ammonia-hydrogen peroxide mixture (APM) solution stripping [12]. Among them, using APM solution to clean thiols is appealing because it has been shown that SAM removal efficiency is as high as 99 % in tens of minutes [12].

In the present study, an Si-based ARROW-B SPR biosensor with a single lithography step was designed, fabricated, and characterized. This is the first work to create an Si-based ARROW-B SPR biosensor with an in situ regeneration process, which we don’t have to realign the optical system and avoid the characterization discrepancy among regenerations. This SPR biosensor has a fluid flow system to transport samples or analytes to the SPR sensing region. In an experiment, 11-MUA and BSA were immobilized on the sensor surface, and APM solution was used to remove them from the sensor surface for in situ regeneration. The output intensity did not significantly change after ten regeneration processes.

## Materials and Methods

### Design and Fabrication of Si-based Waveguide Biosensor

The ARROW-B structure consists of one core and two lower cladding layers. The refractive indices of the core and second cladding layer are both higher than that of the first cladding layer. Light propagates in the core layer via frustrated total internal reflection. A Fabry–Perot cavity is designed within the second cladding layer as a reflector. The antiresonant condition occurs when light encounters destructive interference inside the Fabry–Perot cavity [7]. For an ARROW-B waveguide, the thickness of the second cladding layer is governed by the transverse antiresonant condition, expressed as:

2 $$ d_{2} \cong \frac{\lambda }{{4n_{2} }}\left[ {1 - \left( {\frac{{n_{c} }}{{n_{2} }}} \right)^{2} + \left( {\frac{\lambda }{{2n_{2} \times d_{ce} }}} \right)^{2} } \right]^{{ - \frac{1}{2}}} \left( {2N + 1} \right),\;N = 0,\;1,\;2 \ldots $$

where *N* = 0 in most cases, and parameters *λ*, *n*_2_, *n*_c_, *d*_2_, and *d*_ce_ are the operating wavelength, the refractive index of the second cladding layer, the refractive index of the core layer, the thickness of the second cladding layer, and the effective thickness of the core layer, respectively. Typically, the material of the second cladding layer is designed to be the same as that of the core. Equation (2) can be reduced to [13]:

3 $$ d_{2} \, \cong \, \frac{{d_{ce} }}{2} \, (2N \, + \, 1) $$

Usually, a symmetrical ARROW-B structure was utilized to greatly reduce the propagation loss in the waveguide region resulting from changes in the outermost environment and the scattering loss caused by fabrication imperfections at the core/superstrate boundary [14]. The propagation region includes the front and the rear propagation regions. Then, in this research a symmetrical ARROW-B structure is designed in the propagation regions to isolate the influence of the outermost environment (1.330 ≦ *n*_s_ ≦ 1.450, where *n*_s_ is the refractive index of the superstrate). The front region guides the incident light into the sensing region, and the rear region maintains the light power that carries the sensing message to the output end. Therefore, the front and rear propagation regions need to support an effective single mode in the waveguide, and are required to have stable propagation characteristics as the refractive index of the outermost environment varies.

**Fig. 1:**
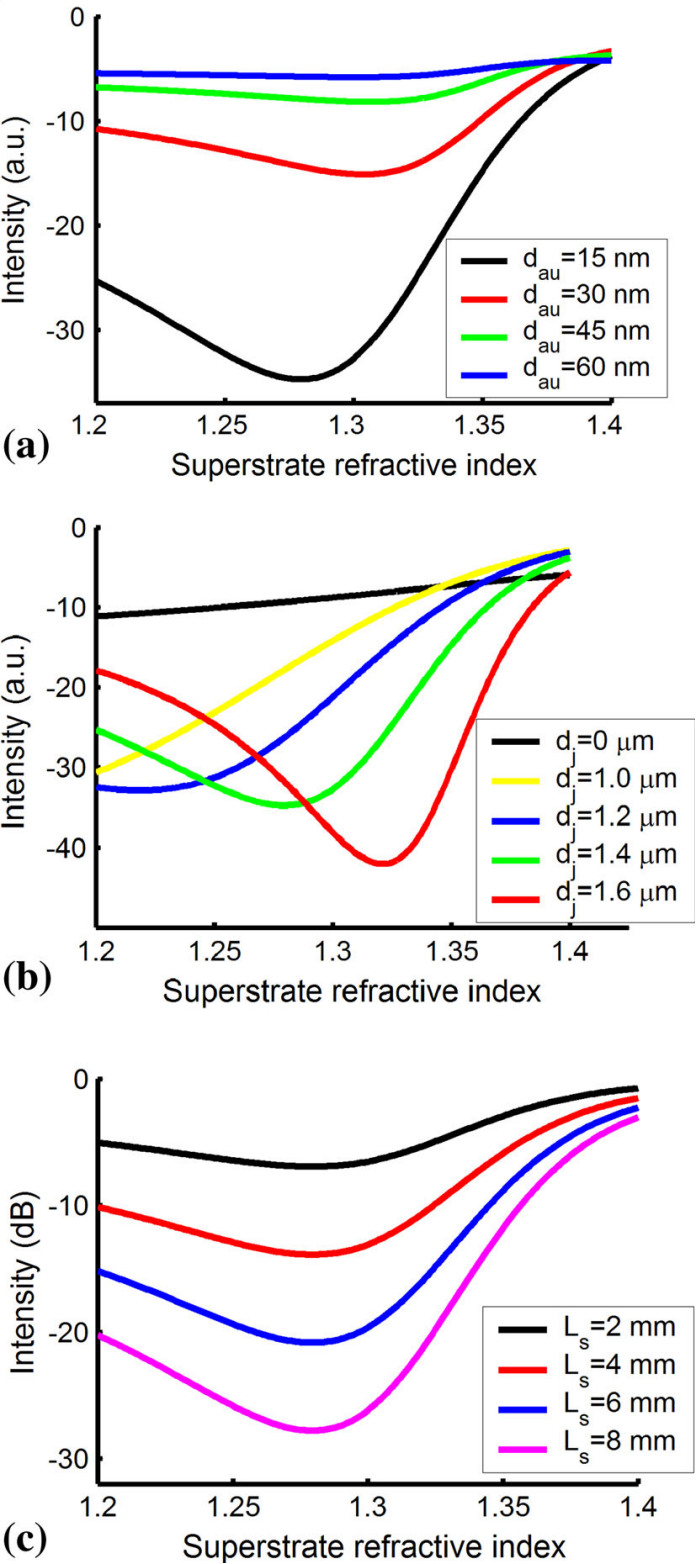
**a** Simulation results of ARROW-B SPR sensor with various gold layer thicknesses. Sensor sensitivity increases with decreasing gold thickness. **b** Simulation results showing adjustment layer thickness shifting SPR dip position. **c** Simulation results showing SPR loss increasing with sensing length

**Fig. 2:**
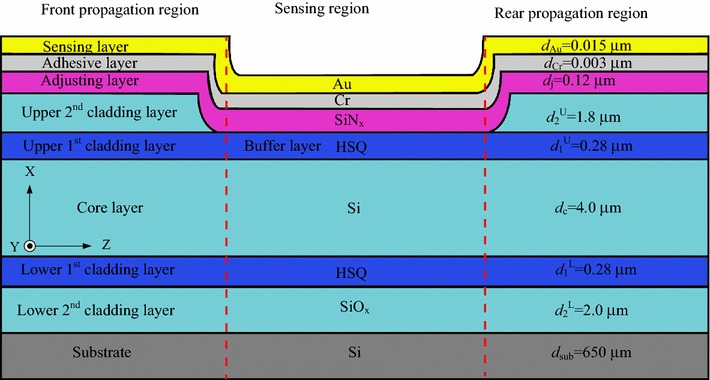
Cross-section of ARROW-B SPR biosensor

In the sensing region, an adjustment layer is directly deposited on the upper first-cladding layer of the ARROW-B waveguide, and then the metal films are evaporated on the adjustment layer. Gold film is widely applied because of its great durability, good corrosion resistance, low electrical resistivity and overall chemical inertness. However, gold film shows poor adherence to dielectric materials [15]. In order to improve adherence between gold and dielectric materials, a chromium layer was utilized. The transfer matrix method was used to simulate the performance of the ARROW-B waveguide structures. As shown in Fig. 1a, the thickness of the gold film (*d*_au_) dominates the sensitivity of the sensor. The thickness of the gold film was chosen as 15 nm for high sensitivity and fabrication reliability. A high-index Si_3_N_4_ adjustment layer was introduced to shift the SPR resonance condition to a different superstrate index. As shown in Fig. 1b, the SPR dip shifts to a larger superstrate index with better sensitivity with increasing Si_3_N_4_ film thickness. Although better sensitivity was obtained when the thickness of the adjustment layer was between 0.14 and 0.16 μm, the thickness of the adjustment layer was chosen as 0.12 μm in order to avoid fabrication errors that deteriorate biosensor performance. In addition, later we show that the sensitivity can be enhanced by increasing the sensing length without changing the superstrate index of the SPR dip. Therefore, the sensing range of the refractive index can be tuned with a fixed adjustment layer. The cross-section of the Si-based ARROW-B sensor is shown in Fig. 2. The front and rear waveguides with upper cladding layers can support low-loss propagation of light even when a high-index O-ring is applied on the surface for biomedical assays. The devices were fabricated using available standard semiconductor processes.

**Fig. 3:**
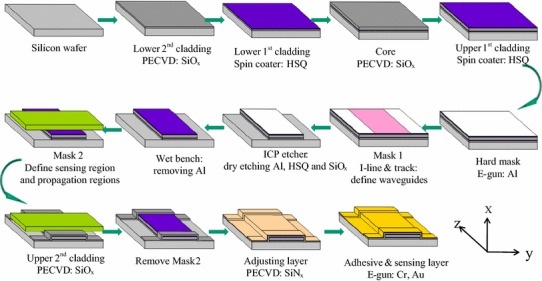
Flow chart of fabrication process for ARROW-B SPR sensor chip

The length of the sensing region should be considered to moderately excite SPWs on the Au surface. If the length of the sensing region is too long, the light will be largely consumed in the Au sensing region, making detection difficult. If the length of the sensing region is too short, the guided mode in the ARROW waveguide could not effectively interact with the SPW at the interface of the metal, and thus the response becomes small. As shown in Fig. 3, the long sensing length resulted in relatively large SPR loss without a shift of the SPR dip. The sensitivity was improved, but measurement equipment with a high resolution was required. Thus, the length of sensing region *L*_s_ was selected as 4 mm to obtain an ARROW-B SPR sensor chip with sufficiently high sensitivity. Also, this sensing length was long enough for transferring the TM-polarized wave to the gold film surface and for exciting SPWs. The lengths of the front and rear regions were also chosen as 4 mm, which were long enough for the wave propagation to be stable before and after the light passed through the sensing region. One advantage of adopting ARROW-B SPR devices is compatibility with current semiconductor processing technology, allowing mass production.

**Fig. 4:**
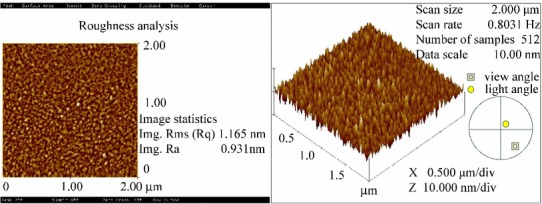
Surface morphology of gold film

**Fig. 5:**
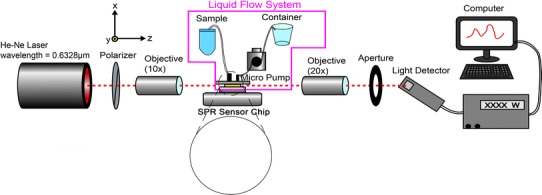
Optical measurement setup with fluid flow system

Figure 4 shows the flow chart of the single-step lithography fabrication processes. In the beginning of chip fabrication, RCA cleaning is performed on 6″ (15.24 cm) silicon wafers to remove organic, native chemical oxide, and ionic contaminants from the wafer surface. Plasma-enhanced chemical vapor deposition (PECVD) was used to deposit a 2-μm-thick SiO_x_ layer as the lower second cladding layer. The thickness was measured using an *n*&*k* analyzer and verified as 2 μm. The refractive index of the film was measured as 1.463 (due to the structure of SiO_x_ instead of pure SiO_2_). Then, a spin-coater was used to spin hydrogen silsesquioxane (HSQ) onto the wafer at 3000 rpm for 30 s. The thickness and refractive index were 0.31 μm and 1.400, respectively, just after spinning. After curing at 350 °C for 15 min, a film with a thickness of 0.28 μm and a refractive index of 1.380 was obtained. PECVD was also used to deposit a 4-μm-thick core layer. Then, repeating the previous step, HSQ was spin-coated to form the upper first-cladding layer for the propagation region and the buffer layer for the sensing region. The lithography process was then used to define waveguide channels. First, Al with a thickness of 3000 Å was deposited via e-gun evaporation as the hard mask. This wafer was put into a track system and an I-line stepper for photoresist coating, exposure, and development. Subsequently, 3000-Å-thick Al and the 6.68-μm-thick SiO_x_/HSQ layers were etched using an inductively coupled plasma dry etcher. Then, residual photoresist and Al were removed using 120 °C H_2_SO_4_/H_2_O_2_ for 10 min using a wet bench. A shadow mask was used to define the sensing region and the propagation regions. After aligning the sensing region of mask 2 to the middle of the channels and using polyimide film tape to fix it, the sensing regions were sheltered, and the uncover regions were the front and rear propagation regions. The upper second cladding layer SiO_x_ on the front and rear propagation regions was deposited on the aforementioned wafer by PECVD. The deposited thickness was 1.80 μm. Subsequently, mask 2 was removed, and a 0.12-μm-thick Si_3_N_4_ layer was deposited using PECVD. An e-gun was used to deposit Cr and Au films with thicknesses of 3 and 15 nm, respectively. Figure 5 shows an AFM image of the fabricated biosensor, which shows that the evaporated gold film was a continuous film with a roughness of 1.165 nm.

### Materials

Sodium chloride solutions of three different concentrations were used to examine the feasibility of the proposed sensor. Phosphate-buffered saline (PBS) consisting of 2.7 mM KCl, 1.5 mM KH_2_PO_4_, 140 mM NaCl, and 8.1 mM Na_2_HPO_4_ at pH 7.2 was used. PBS is isotonic and non-toxic to cells, and thus its primary function is to dilute the biomolecular reagents and to wash off the biomolecular residues in the chamber. 11-MUA was chosen as the SAM material; its concentration was 100 mM. *N*-(3-dimethylaminopropyl)-*N*’-ethylcarbodiimide (EDC) and *N*-hydroxysuccinimide (NHS) (Sigma-Aldrich, USA) were used to activate the terminal carboxylate group of the immobilized 11-MUA. APM solution was used as the regeneration reagent. Hydrogen peroxide (35 %) and ammonia (28 %) (Sigma-Aldrich) were mixed with double distilled (d.d.) water with a volume ratio of 1:1:1. Bovine serum albumin (BSA) is a globular protein, and its precursor contains 607 amino acids with a molecular weight of about 66 kDa. BSA is used in numerous biochemical applications, including the enzyme-linked immunosorbent assay, immunoblots, and immunohistochemistry, due to its stability and lack of interference within biological reactions. The amino group of BSA provides the possibility of combining with carboxylic acid groups, such as 11-MUA. BSA was used to examine the biosensing feasibility in this study.

### Optical Measurement System

**Fig. 6:**
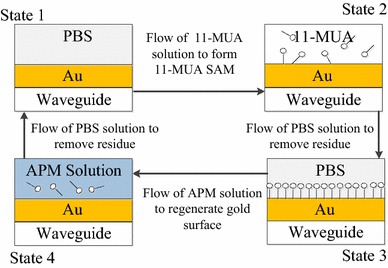
Flow chart of regeneration process

An optical measurement system was set up to perform the bioassay experiments, as shown in Fig. 6. In this optical measurement system, a He–Ne laser with the wavelength 0.6328 μm was used as the light source to excite SPWs in the Au sensing region. Since SPWs can be excited only by TM-polarized waves, the laser beam first passed through a polarizer, and only vertically polarized electric field remained. Then, the vertically polarized light was focused into the input channel of the waveguide by an objective lens (10×). At the output end, the light was collected by an objective lens (20×). Finally, the intensity of the output power was collected by a photodiode. In addition, a fixing stage for the SPR sensor chip was utilized. The freshly cleaned sensor chip was mounted onto this stage so that the input light was able to be focused into one of the waveguides. To construct a seamless liquid flow channel on the sensor chip, an O-ring was added on the chip and sandwiched by two plastic slides. For the fluid flow system, a peristaltic pump was used to propel liquid samples in a plastic tube with a 1.6-mm inner diameter to flow through the sensing region of the chip. The flow system was controlled to have a flow rate of 300 μl/min.

### Regeneration—Removal of 11-MUA from Gold Surface

The principle of the SPR biosensor is based on changes of the superstrate refractive index. Molecular binding on the sensor surface plays an important role in sensing applications. The organic molecule 11-MUA for forming chemical bonds to link analytes and metals or glasses together is frequently used for controlling the adsorption of biomolecules because it offers the opportunity to replace the acid-end group with other functionalized groups quite easily [16]. 11-MUA was immobilized on the gold surface to form a SAM with a thickness of 1.3 nm and a tile angle of 30° from normal [17]. The SAM was stable due to the strong covalent bonds between sulfur and gold atoms. It was difficult to remove this stable thin film because of the strong covalent bond. To regenerate the sensor in situ, APM solution was used. Figure 6 shows the in situ regeneration flow of the SPR sensor. First, PBS solution was used to build the baseline (state 1 in Fig. 6). Then, 11-MUA was flowed onto the surface for 20 h for immobilization (state 2 in Fig. 6). PBS solution was flowed again and the signal difference with the baseline was treated as the SPR response of 11-MUA (state 3 in Fig. 6). APM solution was flowed onto the sensor surface for 2 h to remove the immobilized 11-MUA molecules (state 4 in Fig. 6). After removal of the SAM, PBS solution was used to finish the regeneration process, which returned the sensor to state 1. These steps were repeated to demonstrate the in situ regeneration.

**Fig. 7:**
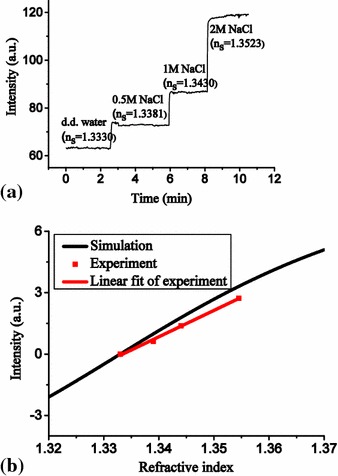
**a** Real-time measurement results of water, and 0.5, 1 and 2 NaCl solutions. **b** Comparison of experimental and simulation results

### Regeneration—Removal of BSA and 11-MUA from Gold Surface

To examine the practicality of the biosensor, BSA was introduced. A mixture solution comprising 10 μM BSA, 0.4 M EDC, and 0.1 M NHS was used after the formation of the 11-MUA SAM, as described previously. With the activation of the 11-MUA by EDC and NHS, the BSA molecules bound to the 11-MUA. We first removed EDC/NHS before BSA immobilization, but the measurement results showed no change after EDN/NHS or BSA solution. Then, the experimental step was modified to include a mixture solution of EDC/NHS/BSA. Then, the measurement results indicated that the BSA or cross-linked BSA molecules were bound with 11-MUA. PBS solution was flowed again to remove residual molecules. The difference between the measured power after BSA and that after 11-MUA corresponded to the consequences of immobilized BSA molecules or cross-linked ones, which was treated as the SPR response of BSA. As in the previous section, APM solution was used to remove 11-MUA and BSA from the surface. PBS solution was flowed and the SPR response was compared with the original baseline to examine regeneration.

## Results and Discussion

NaCl solutions at various concentrations were flowed onto the sensor surface to measure the output power variations for demonstrating the feasibility of the proposed sensor chips. Because the refractive index varies with sodium chloride concentrations, the output power varied with sodium chloride concentrations. The refractive index of d.d. water is 1.3330, and those of 0.5, 1, and 2 M sodium chloride solutions are 1.3381, 1.3430 and 1.3523, respectively. The refractive indices of all sample solutions were measured using a digital refractometer with the precision of ±0.0001. The higher the refractive index of sodium chloride solution, the higher was the detected output power. This tendency agrees with the simulation results. The real-time measurement results and a comparison with the transfer matrix method simulation results are shown in Fig. 7. The sensitivity of the proposed sensor is defined as the ratio of the output power variation to the change of the environment refractive index. The experimental results indicate that the output power was approximately a linear function of the refractive index. The sensitivity of this chip was about 3.0 × 10^3^ μW/RIU. The standard deviation of the output signal of the d.d. water was 0.185 μW and the corresponding resolution was 6.2 × 10^−5^ RIU [18].

**Fig. 8:**
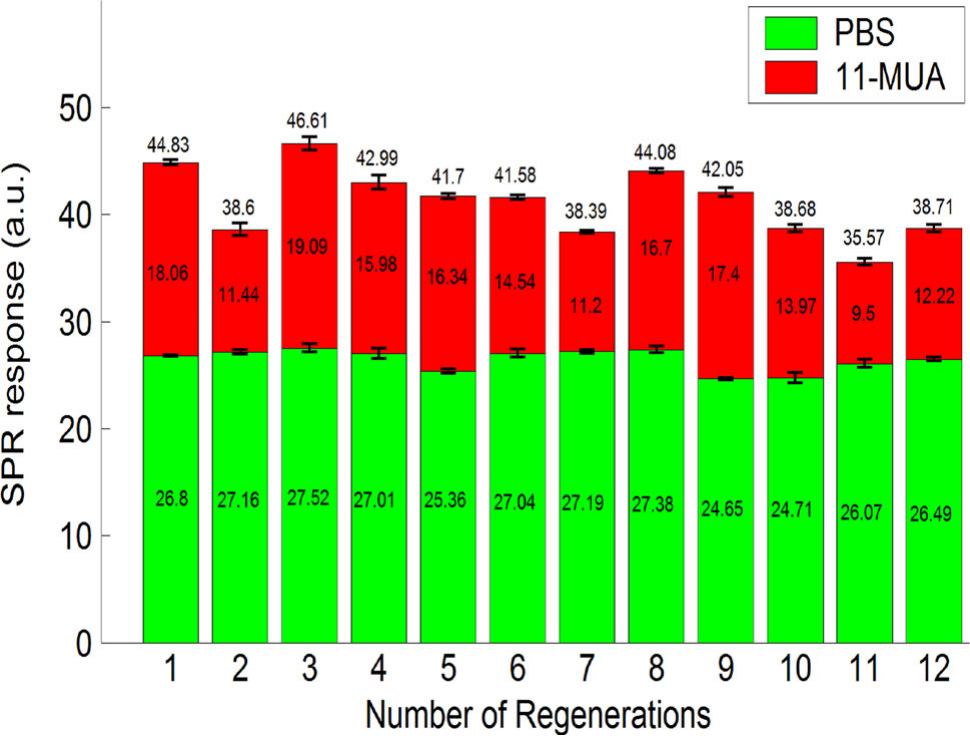
SPR response versus number of regenerations. SPR response of PBS was stable after 12 regenerations

In the experiment, PBS was flowed onto the gold surface to establish a baseline. When the output signal was stable, 100 mM 11-MUA was flowed onto the gold surface and left for 1 day. The output signal increased because the change of the refractive index on the surface resulted from the binding reaction. This binding reaction is the sulfur of MUA binding on the Au surface. PBS was flowed again to wash off the MUA residue from the chip surface. The output signal decreased but was still higher than the PBS baseline. This difference showed the immobilization of SAM on the gold surface. The regeneration reagent solution was then flowed onto the surface for 2 h to remove the immobilized MUA. PBS was flowed to wash off the residual regeneration reagent. The output signal after the reaction of the regeneration reagent was the same as that before SAM formation, which means that this regeneration reagent could completely remove 11-MUA from the gold surface.

**Fig. 9:**
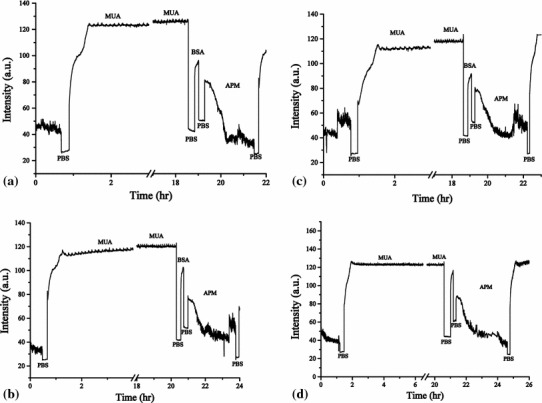
**a**–**d** SPR responses of four real-time regenerations

**Fig. 10:**
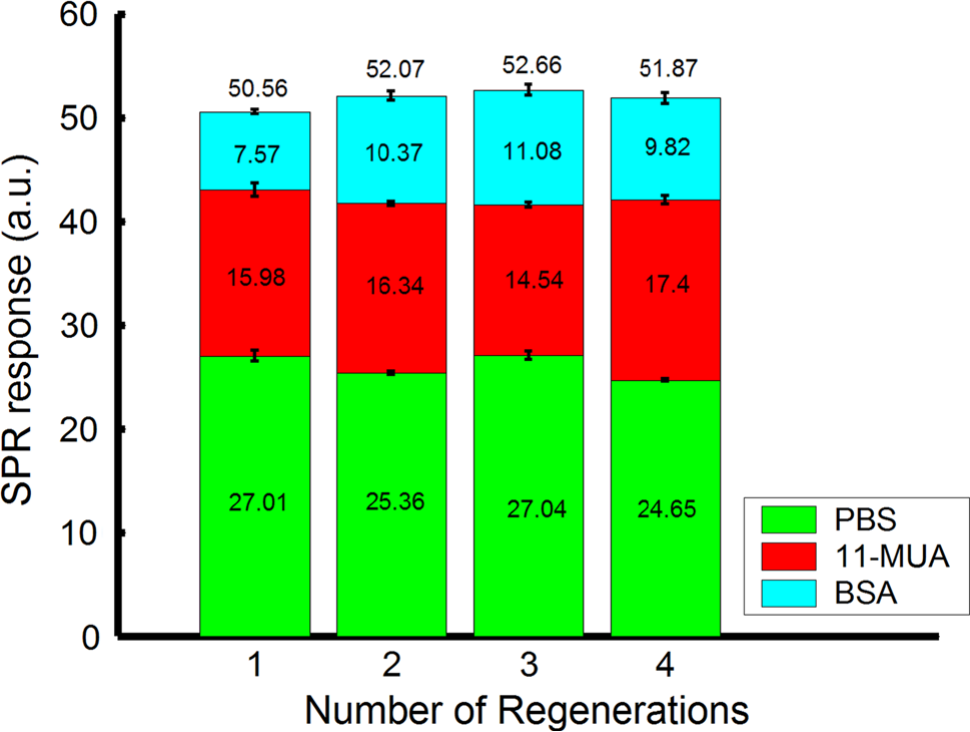
SPR responses of PBS, 11-MUA, and BSA in four regenerations

The results of repeated experiments of the removal of 11-MUA from the gold surface are shown in Fig. 8. The SPR responses of the PBS baseline and 11-MUA were 26.45 ± 1.02 and 14.70 ± 3.06 μW, respectively. Though the SPR responses of 11-MUA showed some variation, which might have resulted from inaccurate experiment control of 11-MUA binding, the variation of SPR responses of the PBS baseline was less than 4 % after 12 regenerations, confirming the reusability of this sensor. Figure 9 shows real-time measurement results of four regenerations with 11-MUA and BSA binding. In each regeneration, the SPR responses in the PBS baselines after 11-MUA and BSA bindings are clearly changed. The numerical analysis of Fig. 9 is shown in Fig. 10. The SPR response of BSA is 9.71 ± 1.52 μW. The results show the capability of biomolecular immobilization on the sensor. Therefore, the reusability of this sensor was verified. This reusable ARROW-B SPR biosensor can also detect other biomolecules being able to be bounded with the 11-MUA, e.g., peptides and proteins.

## Conclusion

This study described the design, fabrication, and characterization of an Au-coated ARROW-B SPR biosensor. Regeneration experiments were conducted using APM solution to verify reusability. The Au-coated SPR biosensor allows label-free, real-time detection with high sensitivity and great reliability in aqueous environments. The proposed ARROW-B SPR biosensor is compatible with the standard semiconductor fabrication process so that lab-on-chip can be easily realized in the future. Single-step lithography is used to simplify the fabrication process.

The feasibility of the proposed ARROW-B SPR biosensor was demonstrated. The intensity interrogation method was used to measure sodium chloride solution with various concentrations. The sensitivity of this SPR sensor was 3.0 × 10^3^ μW/RIU with a resolution of 6.2 × 10^−5^ RIU. Furthermore, bioassay experiments of SAM formation and BSA binding reaction were conducted. The binding reaction was between the amino group of BSA and the terminal carboxylate group of 11-MUA, which was immobilized on the gold surface and activated by EDC and NHS. The measurement results demonstrated the output power changed with the different bimolecular layer on the metal surface as a result of binding reactions owing to the environment refractive index change. An APM solution with a volume ratio of 1:1:1 was used to remove thiols from the gold surface to regenerate the biochip in situ. The regeneration process takes about 2 h. The regeneration experiments were repeated to confirm the reusability of the ARROW-B SPR biosensor. Experimental results of regeneration showed approximately the same PBS baseline responses after the sensor was reused more than 10 times.

## References

[1] Homola J, Yee SS, Gauglitz G (1999). Surface plasmon resonance sensors: Review. Sensors and Actuators B: Chemical.

[2] Nylander C, Liedberg B, Lundstrom I (1983). Surface plasmons resonance for gas detection and biosensing. Sensors Actuator.

[3] Chung JW, Kima SD, Bernhardt R, Pyun JC (2005). Application of SPR biosensor for medical diagnostics of human hepatitis B virus (hHBV). Sensors and Actuators B: Chemical.

[4] Piliarik M, Parova L, Homola J (2009). High-throughput SPR sensor for food safety. Biosensors & Bioelectronics.

[5] Homola J, Koudela I, Yee SS (1999). Surface plasmon resonance sensors based on iffraction gratings and prism couplers: Sensitivity comparison. Sensors and Actuators B: Chemical.

[6] Duguay MA, Kokubun Y, Koch TL, Pfeiffer L (2001). Antiresonant reflecting optical waveguides in Si-O_2_-Si multilayer structures. Applied Physics Letters.

[7] Baba T, Kokubun Y (1989). New polarization-insensitive antiresonant reflecting optical waveguide (ARROW-B). IEEE Photonics Technology Letters.

[8] Ostuni E, Yan L, Whitesides GM (1999). The interaction of proteins and cells with self-assembled monolayers of alkanethiolates on gold and silver. Colloids and Surfaces B: Biointerfaces.

[9] Vericat C, Vela ME, Benitez G, Carro P, Salvarezza RC (2010). Self-assembled monolayers of thiols and dithiols on gold: New challenges for a well-known system. Chemical Society Reviews.

[10] Canaria CA, So J, Maloney JR, Yu CJ, Smith JO, Roukes ML, Fraser SE, Lansford R (2006). Formation and removal of alkylthiolate self-assembled monolayers on gold in aqueous solutions. Lab on a Chip.

[11] Worley CG, Lintona RW (1995). Removing sulfur from gold using ultravioletozone cleaning. Journal of Vacuum Science and Technology A.

[12] Kim DJ, Pitchimaini R, Snow DE, Hope-Weeks LJ (2008). A simple method for the removal of thiols on gold surfaces using an NH_4_OH-H_2_O_2_-H_2_O solution. Scanning.

[13] Baba T, Kokubun Y (1992). Dispersion and radiation loss characteristics of antiresonant reflecting optical waveguide-numerical results and analytical expressions. IEEE Journal of Quantum Electronics.

[14] Huang, C. C., Hsu, H. F., Chen, S. H., Tsai, K. Y., Huang, Y. T., Lin, C. S., & Hsu, S. H. (2012). Real-time detection of α-torombin binding to single-strand DNA aptamers by a highly sensitive Si-based waveguide SPR biosensor. *The 3rd Asia*-*Pacific Optical Sensors Conference 2012 (APOS2012)*, Sydney, Australia.

[15] Yoo KS, Sorensen IW, Glaunsinger WS (1994). Adhesion, surface morphology, and gas sensing characteristics of thin gold file chemical sensors. Journal of Vacuum Science and Technology A.

[16] Frey BL, Corn RM (1996). Covalent attachment and derivatization of poly (l-lysine) monolayers on gold surfaces as characterized by polarization-modulation FTIR spectroscopy. Analytical Chemistry.

[17] Bain CD, Evall J, Whitesides M (1999). Formation of monolayers by the coadsorption of thiols on gold: Variation in the head group, tail group, and solvent. Journal of the American Chemical Society.

[18] Homola J (2008). Surface plasmon resonance sensors for detection of chemical and biological species. Chemical Reviews.

